# What predicts negative effects of rheumatoid arthritis? A follow-up two years after diagnosis

**DOI:** 10.1186/2193-1801-3-118

**Published:** 2014-02-28

**Authors:** Catharina Gåfvels, Margareta Hägerström, Birgitta Nordmark, Per Wändell

**Affiliations:** Department of Social Work, Karolinska University Hospital, Solna, 171 76 Stockholm Sweden; Centre for Family Medicine, Karolinska Institutet, Alfred Nobels allé 12, Huddinge, 141 83 Sweden; Department of Rheumatology, Karolinska University Hospital, Solna, 171 76 Stockholm Sweden

**Keywords:** Rheumatoid arthritis, Psychosocial factors, Adaptation, Anxiety, Depression, Coping

## Abstract

We aimed at analyzing important predictive factors for experienced negative emotional and social effects of rheumatoid arthritis (RA) two years after diagnosis in patients aged 18–65 years. The first group included 41 participants, who had psychosocial problems (PSP) already at diagnosis, and who received an intervention by a medical social worker to improve coping capacity and social situation. The second group included 54 patients (NPSP) without such problems at diagnosis. All completed a questionnaire mapping their social situation, the Hospital Anxiety and Depression Scale (HADS), the Sense of Coherence Scale (SOC) and the General Coping Questionnaire (GCQ) at diagnosis and after 24 months. The most pronounced predictive factor for a strong impact of the disease was high scores on HADS depression scale. After 24 months, PSP participants had a more strained life situation, with higher scores on anxiety and depression and lower on SOC, in comparison with NPSP. NPSP participants improved their coping strategies regarding self-trust, cognitive revaluation, protest and intrusion, but deteriorated regarding problem focusing and social trust. PSP patients kept their initial coping strategies, except for intrusion decreasing over time, and seemed to have a more rigid coping pattern. However, the experienced negative impact of the disease increased over time in both groups despite improvement in sickness related data. Mostly influenced areas were economy, leisure time activities and social life. We conclude that psychosocial consequences of RA are more connected to emotional and social vulnerability than are RA-related clinical factors.

**Electronic supplementary material:**

The online version of this article (doi:10.1186/2193-1801-3-118) contains supplementary material, which is available to authorized users.

## Introduction

Rheumatoid arthritis (RA) is a common chronic disorder, affecting nearly 0.5% of the Swedish population (Simonsson et al. 1999), with an estimated annual incidence among women at 0.29‰ vs. 0.18‰ among men (Soderlin et al. 2002). Negative consequences of a chronic disease such as RA could be of both somatic and psychosocial origin. Even though medical treatment has improved over time, knowledge of psychosocial consequences of RA is limited. RA is associated with socio-economic changes even at early stages of the disease (Albers et al. 1999), including work disability (Sokka 2003), and psychosocial problems are present almost in half of the patients already at diagnosis (Gafvels et al. 2012). These problems are often associated with depression and functional status (Morris et al. 2008), with difficulties at work appearing even at early phases (Geuskens et al. 2007). The risk of depression is higher in early stages of RA (Treharne et al. 2005), and findings in a review article do suggest that psychological interventions may be more effective for patients with a shorter duration of the disease (Astin et al. 2002). Low social support is considered a predictor of depression and anxiety in people with RA (Zyrianova et al. 2006), and high social support might weaken the effects of distress, at least in people at an early stage of RA (Strating et al. 2006).

Coping is a theoretical concept often used when discussing a person’s ability to adapt to stressors in general (Lazarus and Folkman 1984), but it is also commonly used to describe a person’s adaptation to a chronic disease (Walker et al. 2004; Persson et al. 1999). One definition of coping is “constantly changing cognitive and behavioural efforts to manage specific external and/or internal demands that are appraised as taxing or exceeding the resources of a person” (Lazarus and Folkman 1984). Active coping could be defined as “efforts to act on the source of stress or one’s emotions” and passive coping as “efforts to avoid or deny the stressor” (Compas et al. 2012). Studies have shown that patients with RA using passive coping strategies are at risk of developing psychological co-morbidity (Treharne et al. 2007), and to have more pain (Covic et al. 2000). However, in general it is unclear which coping strategies leads to psychological distress in later in the course of RA (Vriezekolk et al. 2011). Another concept related to coping is “Sense of coherence” (SOC) (Antonovsky 1993), focusing on health-preserving factors in counteracting negative consequences of psychosocial stress (Schnyder et al. 1999). The concept of SOC deals with an individual’s capacity to cope with psychosocial stress. Protective factors measured by the scale are comprehensiveness, meaningfulness, manageability, and resistance resources.

The aim of this study was to investigate predictive factors for negative effects of RA by studying the psychosocial situation 24 months after diagnosis in two groups of RA patients. The hypothesis was that a vulnerable psychosocial situation would predict a negative effect of RA at the follow-up.

## Materials and methods

### Setting and participants

This paper is based on a prospective study on psychosocial consequences of RA and diabetes mellitus (Gafvels et al. 2012; Rane et al. 2011). Measurements were performed at inclusion and after 24 months. The RA participants were patients from the Department of Rheumatology, Karolinska University Hospital, recruited from the beginning of 2001 to the end of 2004. All patients fulfilling the inclusion criteria: with a new diagnosis (not older than three months) of RA, according to the American College of Rheumatology (ACR) 1987 classification criteria (Levin et al. 1996); aged 18 to 65 years; and able to communicate in Swedish; were invited to participate in the study. Altogether 123 patients (90 women) were eligible and received verbal and written information about the study. However, 19 of these declined to participate before the study started, and four dropped out during the data collection (two died, one was diagnosed with cancer and the fourth declined during the first part of the data collection). These 23 patients (15 women) were excluded from the study (Gafvels et al. 2012). No significant differences were found as regards age and sex between the participants and non-participants. The participants were divided into two groups based on a semi-structured interview, which consisted of a psychosocial mapping, serving as a basis of psychosocial diagnosis, according to praxis in social work in health care (Gafvels et al. 2012). The first group (n = 41) consisted of patients with psychosocial problems (PSP) who received intervention by the medical social worker in the study. Five patients with psychosocial problems did not want to pay visits to the hospital social worker, and were followed up by telephone calls every sixth month. As they did not receive any intervention they were excluded from the analysis. The second group (n = 54) consisted of patients with no pronounced psychosocial problems (NPSP).

### Outcomes

The main outcome variable was the experienced negative effect of the disease two years after the diagnosis. Response alternatives were: 1) little or not at all, 2) moderate and 3) great or very great, which we dichotomized into great or very great negative effect, i.e. response alternative 3, versus not, i.e. response alternatives 1 and 2. Another question with the same response alternatives registered expected consequences at baseline (Gafvels et al. 2012). Information of influenced areas of life was also collected.

### Categorization of psychosocial problems

Psychosocial problems were classified as: a) strongly negative, or inadequate, psychological reactions to the disease; b) existing difficult social and/or psychological life conditions with no direct relationship to RA; or c) strained social and/or psychosocial conditions difficult to combine with adaptation to RA. Goals for intervention by the medical social worker were: a) to strengthen the patients’ capacity to cope with their problems and b) to affect the patients’ social situation positively according to Swedish practice for psychosocial work in healthcare (Gafvels et al. 2012). An intervention goal for each patient was set according to his/her problems or needs. The sessions lasted for one hour, and in mean the patients had 10 sessions each during a three months’ period.

### Measurements

Social situation, i.e. family, education, employment, housing, certain life events, life style habits, social network and support, was measured by a questionnaire from the Epidemiological Investigation on Rheumatoid Arthritis study (EIRA) exploring environmental and genetic risk factors in the development of RA that started in 1997 (Bengtsson et al. 2009). Social support was measured with questions regarding support from workmates, and from people outside family and work. Questions about attitudes to, knowledge about and consequences of RA, were included in the questionnaire. Educational level was defined as the highest completed level reached, i.e. compulsory school, high school, or university.

The Hospital Anxiety and Depression Scale (HADS), which was developed by Zigmond and Snaith to detect anxiety and depression in patients with somatic conditions (Zigmond and Snaith 1983), was used to measure anxiety and depression at baseline and at 24 months. The HADS has been evaluated in a Swedish population (Lisspers et al. 1997). Cronbach’s alpha for HADS-A was 0.84, and for HADS-D 0.82. The questionnaire is self-administered and consists of two subscales (anxiety and depression) with 7 items, each rated on a 4-point Likert scale from “no” to “maximum”. According to Zigmond and Snaith, items are summed into a dimensional score for anxiety and depression, respectively, with ≤ 7 indicating “no case”, 8–10 “possible case”, and ≥11 “probable case”.

The 13-item Sense of Coherence (SOC) scale, developed and modified by Antonovsky, measures attitudes and resources for handling psychosocial stress (Antonovsky 1993). Important components measured by the scale include comprehensiveness, meaningfulness, manageability, and resistance resources. The scale should be treated as a single entity with no subscales, and the values may vary between 13 and 91. The higher the SOC scores, the better the ability to cope with stress. Cronbach’s alpha in SOC-13 was 0.74–0.91 in performed studies (Antonovsky 1993).

Coping strategies were measured with the General Coping Questionnaire (GCQ). This questionnaire was based on Lazarus’ model (Lazarus and Folkman 1984), further developed and evaluated by Persson (Persson et al. 2013), and has previously been used in studies of patients with diabetes (Gafvels and Wandell 2006,2007). The instrument is divided into five coping orientations dichotomized into positive and negative opposites; i.e., self-trust/fatalism, problem-focusing/resignation, cognitive revaluation/protest, social trust/isolation, and minimization/intrusion. The 10 items are rated between 0 and 100, with 0 signifying the lowest and 100 the highest possible score on the specific scale, i.e. 100 is the best positive on the positive scales and the worst possible on the negative ones. Cronbach’s alpha for the different scales was 0.74–0.91 (Persson et al. 2013).

### Collection of psychosocial data

Participants filled in the questionnaires, i.e. social situation (EIRA), depression and anxiety (HADS), and coping attitude and strategies (SOC and GCQ), at baseline, i.e. within 3 months after diagnosis, and 24 months after diagnosis. Upon inclusion, an experienced medical social worker not involved in the study performed an interview using a structured form (Additional file 1), described in our earlier paper of the baseline examination of the sample (Gafvels et al. 2012).

### Collection of clinical data

Data were derived from the Swedish Rheumatology Quality Register (SRQ) (Askling et al. 2006), and are based on assessments by a rheumatology team of the Karolinska Hospital. The team consists of a rheumatologist, a nurse, a medical social worker, a physiotherapist and an occupational therapist. The severity of the disease was assessed by the Disease Activity Score for 28 joints (DAS 28) (Prevoo et al. 1995), Health Assessment Questionnaire (HAQ) (Ekdahl et al. 1988), the patients Visual Analogue Scale (VAS) for global perceived disease activity assessed during the last week (scores 0–100), i.e. “How have you been the last week, in general, given your arthritis?”, with 0 signifying “entirely good” and 100 “as bad as can be imagined”, and joint pain (Kalyoncu et al. 2009), and finally the rheumatologist assessment score of 0–5 for overall disease activity. DAS 28 is based on both patient scoring of global disease impact and clinical data including a score of tender and swollen joints, and the erythrocyte sedimentation rate (ESR). HAQ evaluates daily activity functions of the patients, with difficulties in 8 domains such as dressing, arising, eating, walking, hygiene, reaching, gripping, and other activities. Along with the core set DAS 28 a rheumatologist assessed the over-all disease activity with a 5-graded Likert scale (1: no disease activity; 2: low disease activity; 3: moderate disease activity; 4: high disease activity; and 5: maximal disease activity). Medical treatment was initiated at the time of diagnosis. The studied patients’ pharmacological treatment was not known, but since all these patients belonged to an early arthritis clinic we can assume that the pharmacological treatment was according to the guidelines for early rheumatoid arthritis. The SRQ Register report that the initial treatment for early rheumatoid arthritis in Stockholm the years 2001–2004 was Methotrexate 54–70%, Sulphasalazine 21–11%, biologics 3,5–4%, combinations 1,5–5%, others 4,5–2%. 16–7% were not treated initially (Askling et al. 2006). There is no report on how many patients were treated with low-dose corticosteroids in the Register but this was done quite often, as well as treatment with intra-articular corticosteroid injections.

### Statistical analysis

Results were analyzed in crosstabs and multivariate analyses. First, we compared differences within groups over time and between groups. Statistical methods used to calculate significant differences were Chi-square, Fisher’s exact test, Wilcoxon’s paired test, and Mann Whitney’s test. Secondly, we present results from the HADS, SOC, and GCQ scales as median values with interquartile ranges because of significant skewness. The level of statistical significance was set at p <0.01, due to multiple comparisons, but in change over time at p <0.05. Thirdly, we performed age- and sex-adjusted logistic regression models for each factor of interest, using the effects of the disease experienced at 24 months, dichotomized as great versus not, as the dependent variable. As independent factors we tested the social background variables, emotional status, coping strategies, disease-related data and self-reported reaction data at baseline. Fourthly, we performed a multivariate analysis, again using effects of the disease experienced at 24 months as the dependent variable, with adjustment for age and sex and including factors with p < 0.1. We tested model specification and Goodness-of-fit by Hosmer-Lemeshow’s test.

### Ethical considerations

This study was approved by the Research Ethics Committee at Karolinska Institutet (No. 00–065). Information to the participants was given by the nurse at the early arthritis clinic at the Department of Rheumatology, and participants gave their oral consent before responding to the questionnaires. ClinicalTrials.gov. identifier: NCT01066130.

## Results

Self-reported demographic and social background data are shown in Table 1. Most patients in each group were women, without significant difference in distribution between the groups, 83% in the PSP group vs. 70% in the NPSP group. The differences in social situation between PSP and NPSP patients were unchanged from baseline to follow-up, i.e. the PSP patients were younger, more often unmarried, divorced, or widowed, more often reported a strained financial situation, and more often were on long-term sick-leave or early retirement pension. PSP patients also reported less affinity with workmates both at baseline and at 24 months, but lower support from workmates only at 24 months. As regards the type of psychosocial problems in the PSP group, 15 (37%) had problems solely as a consequence of their RA, 9 (22%) had problems in their life situation in general not connected to RA, and for the remaining 17 (42%) problems were related to RA in combination with an already strained life situation.

**Table 1:** **Self-reported demographic and social background of patients 20–65 years who participated in this study on adaptation to newly diagnosed rheumatoid arthritis (n = 95)**

	PSP, baseline	PSP, 24 months	Diff within group	NPSP, baseline	NPSP, 24 months	Diff within group	Diff at baseline	Diff at 24 months
	(n = 41)			(n = 54)				
Men	7 (17%)	–	–	16 (30%)	–	–	0.16	–
Women	34 (83%)	–	–	38 (70%)	–	–		–
Mean age, years (SD)	44.4 (11.2)			50.4 (10.5)			0.0060	
Born in Sweden	35 (85%)	–	–	47/52 (90%)	–	–	0.46	–
Marital status:			0.96			0.91	<0.001	<0.001
Married	13 (32%)	12 (29%)		39 (72%)	41 (76%)			
Unmarried	15 (37%)	15 (37%)		8 (15%)	7 (13%)			
Divorced/widowed	13 (32%)	14 (34%)		7 (13%)	6 (11%)			
Living with partner	21 (51%)	18 (44%)	0.29	42 (78%)	42 (78%)	1.00	0.018	0.003
Living alone	17 (42%)	15 (37%)		9 (17%)	9 (17%)			
Living with children	23/39 (59%)	19 (48%)	0.26	21 (39%)	19/53 (36%)	0.75	0.056	0.30
Educational level:							0.31	–
Compulsory school	12 (32%)	–	–	16 (31%)	–	–		–
High school	15 (41%)	–	–	14 (28%)	–	–		–
University	10 (27%)	–	–	21 (41%)	–	–		–
Employed	22 (54%)	17 (41%)	0.31	42 (78%)	39 (72%)	0.080	0.004	0.005
On early pension/long-term sick-leave	14 (34%)	21 (51%)		4 (7%)	11 (20%)			
Other	5 (12%)	3 (7%)		8 (15%)	4 (7%)			
Difficult financial situation	10 (24%)	14 (34%)	0.94	0 (0%)	2 (4%)	0.50	<0.001	<0.001
Financial problems*	10 (24%)	18 (44%)	0.062	2 (4%)	2 (4%)	1.00	0.004	<0.001
Own their residence	20 (74%)	21 (51%)	0.83	36 (67%)	35 (65%)	0.84	0.079	0.18
Social support:								
From workmates	19/33 (58%)	17/33 (52%)	0.62	30/46 (65%)	36/48 (75%)	0.30	0.48	0.029
Outside family and work	35 (85%)	34 (83%)	0.76	46/52 (89%)	51/54 (94%)	0.32	0.66	0.095
Affinity with workmates	18/33 (55%)	20/34 (59%)	0.72	37/46 (80%)	43/48 (90%)	0.21	0.014	0.001
Good relationship with ≥ 3 workmates	10/33 (30%)	16/33 (48%)	0.13	23/47 (49%)	32/48 (67%)	0.080	0.096	0.10

Figures at baseline and after 24 months.

(Results given as numbers and percentages unless otherwise is stated).

*Have had difficulties paying bills during the last 12 months.

PSP = Patients with psychosocial problems.

NPSP = Patients with no psychosocial problems.

Table 2 shows data on the participants’ psychological state as regards anxiety, depression, sense of coherence, and coping strategies. The PSP patients had higher anxiety and depression scores and lower SOC scores than had NPSP patients on both occasions. With respect to coping strategies, PSP patients showed significantly higher, i.e. worse, scores on protest, isolation and intrusion, and lower scores, i.e. worse, on minimization, on both occasions. Differences found only at 24 months concerned: self-trust, with a lower score (i.e. worse); fatalism, with a higher score (i.e. worse); and social trust, with a higher score (i.e. better) in the PSP group. Concerning changes over time, the PSP group had a decreased score only for intrusion (i.e. improved). For the NPSP group there were several significant changes over time: increased scores for self-trust and cognitive revaluation (i.e. improved); decreased scores for protest, and intrusion (i.e. improved); and decreased scores for problem-focusing and social trust (i.e. worse). By means of comparing the groups’ Δ values over time, the only significant difference found was in self-trust, which was higher, i.e. better, in the NPSP group than the PSP group.

**Table 2:** **Emotional status and coping strategies of patients 20–65 years who participated in this study on adaptation to newly diagnosed rheumatoid arthritis (n = 95)**

	PSP, baseline	PSP, 24 months	Diff within group	NPSP, baseline	NPSP, 24 months	Diff within group	Diff of Δ-values between groups	Diff at baseline	Diff at 24 months
	(n = 41)		p-values*	(n = 54)		p-values*	p-values*	p-values*	p-values*
HADS scores									
Anxiety	9.0 (5.5–12.0)	8.0 (4.5–12.0)	0.24	4.0 (2.0–7.3)	3.0 (1.0–5.0)	0.008	0.57	<0.001	<0.001
Depression	6.0 (4.0–8.5)	5.0 (2.0–9.0)	0.42	3.0 (1.0–5.0)	2.0 (1.0–4.3)	0.033	0.69	<0.001	<0.001
SOC scores	62 (56–70)	60 (50–70)	0.040	73 (66–81)	71 (66–80)	0.56	0.90	<0.001	<0.001
Low SOC	12 (29%)	23 (56%)	0.039	11 (20%)	8 (15%)	0.51		0.60	<0.001
Moderate SOC	18 (44%)	13 (32%)		27 (50%)	24 (45%)				
High SOC	11 (27%)	5 (12%)		16 (30%)	21 (40%)				
Coping strategies (GCQ)									
Self-trust	80 (60–85)	75 (60–85)	0.24	82.5 (70–90)	85 (75–100)	0.012	0.012	0.23	<0.001
Fatalism	30 (20–35)	30 (20–40)	0.28	20 (10–35)	20 (5–34)	0.66	0.27	0.064	0.0054
Problem focusing	85 (75–95)	85 (60–90)	0.15	88 (80–95)	80 (68–95)	0.008	0.50	0.61	0.87
Resignation	20 (10–25)	17.5 (1.3–23.8)	0.28	10 (0–20)	15 (0–25)	0.14	0.08	0.024	0.93
Cognitive revaluation	40 (25–60)	45 (30–70)	0.13	35 (25–50)	55 (40–79)	0.004	0.54	0.69	0.30
Protest	40 (25–65)	40 (20–65)	0.95	20 (6–34)	12.5 (5–24)	0.008	0.29	<0.001	<0.001
Social trust	87 (75–100)	87 (67–98)	0.19	90 (78–100)	80 (60–87)	<0.001	0.17	0.77	<0.001
Isolation	20 (12–40)	28 (16–36)	0.44	8 (0–16)	12 (4–16)	0.97	0.53	<0.001	<0.001
Minimization	72 (56–80)	68 (56–80)	0.68	84 (65–92)	80 (72–88)	0.83	0.76	0.0053	0.0010
Intrusion	40 (25–56)	30 (20–50)	0.026	20 (10–39)	15 (5–24)	0.001	0.55	<0.001	<0.001

Results presented as medians (interquartile range).

*P values analyzed within groups by Wilcoxon’s paired test, and between groups Mann–Whitney test.

PSP = Patients with psychosocial problems.

NPSP = Patients with no psychosocial problems.

Table 3 shows that disease-related activity, bothself-reported and physician-assessed, decreased significantly over time in both groups. Scores for self-reported global perceived disease activity and pain were higher in the PSP group both at baseline and at follow-up. When comparing PSP patients (n = 24) who had achieved their psychosocial treatment goals after 24 months to those who had not (n = 16), significantly lower scores for global perceived disease activity at follow-up were found, 24.5 vs 48.0 (p = 0.001). As regards global perceived disease activity at baseline, there was no significant difference according to the chosen p-level of 0.01 (median 58.0 vs 68.5 (p = 0.014). When PSP patients’ problems were stratified as a) problems caused by the disease (n = 15), b) difficult social and/or psychological life conditions not caused by RA (n = 9) or c) a combination of both (n = 17), their median sickness scores at baseline compared to at follow-up did significantly change in subgroups a) (59 vs 27, p = 0.008) and c) (67 vs 42, p = 0.021), but not in b) (55.0 vs 45.5, p = 0.58).

**Table 3:** **Clinical data on patients 20–65 years who participated in this study on adaptation to newly diagnosed rheumatoid arthritis (n = 95)**

Variable	PSP, baseline	PSP, 24 months	Diff within group	NPSP, baseline	NPSP, 24 months	Diff within group	Diff of Δ-values between groups	Diff at baseline	Diff at 24 months
	(n = 41)		p-values*	(n = 54)		p-values*	p-values*	p-values*	p-values*
Other important disease	16 (39%)			24 (45%)				0.54	
DAS-28	5.67 (4.72–6.36)	3.30 (2.45–4.34)	<0.001	5.09 (49–5.66)	3.08 (2.18–3.86)	<0.001	0.39	0.023	0.26
HAQ	1.13 (0.75–1.50)	0.63 (0.38–1.00)	<0.001	1.00 (0.63–1.25)	0.25 (0.00–0.63)	<0.001	0.11	0.15	<0.001
Patient-reported global perceived disease activity score	64 (42–77)	37 (20–67)	<0.001	44 (24–67)	21 (7–49)	<0.001	0.62	0.0024	0.019
Patient-reported pain	62 (45–71)	29 (16–52)	<0.001	48.5 (28–66)	18 (5–50)	<0.001	0.89	0.028	0.011
Disease activity score (assessed by rheumatologist)	3.0 (3.0–4.0)	2.0 (1.3–2.0)	<0.001	3.0 (3.0–3.0)	2.0 (1.0–2.0)	<0.001	0.59	0.59	0.43

Results presented as medians (interquartile range). Comparing data at diagnosis (or at 6 months) with at 24 months (or at 18 months).

*P values analyzed within groups by Wilcoxon’s paired test, and between groups Mann–Whitney test.

PSP = Patients with psychosocial problems.

NPSP = Patients with no psychosocial problems.

Table 4 shows self-reported reactions to RA. Experiences at 24 months appeared worse than expectations at baseline for both groups. In regard to social and economic consequences of RA, significantly more PSP patients reported an effect on all areas of interest with exception of work. More PSP patients than NPSP patients reported that they had given up leisure activities because of their symptoms both at baseline and 24 months.

**Table 4:** **Self-reported reaction to the diagnosis in patients 20–65 years who participated in this study on adaptation to newly diagnosed rheumatoid arthritis (RA) (n = 95)**

	PSP, baseline	PSP, 24 months	Diff within group	NPSP, baseline	NPSP, 24 months	Diff within group	Diff at baseline	Diff at 24 months
	(n = 41)		p-values*	(n = 54)		p-values*	p-values*	p-values*
Sufficient knowledge about RA		31 (78%)			39 (75%)			0.78
Insufficient knowledge		9 (23%)			13 (25%)			
Impact of the disease:			<0.001			0.002	0.014	<0.001
Not at all or little	6 (15%)	0 (0%)		12 (23%)	5 (9%)			
Moderately	23 (58%)	12 (29%)		38 (72%)	32 (60%)			
A lot	11 (28%)	29 (71%)		3 (6%)	16 (30%)			
Influenced areas:								
Relationship to partner	–	16/39 (41%)	–	–	7/43 (16%)	–	–	0.013
Sexual life	–	15/39 (39%)	–	–	8/44 (18%)	–	–	0.039
Family life	–	21/39 (54%)	–	–	10/42 (24%)	–	–	0.005
Social life	–	24/38 (63%)	–	–	13/42 (31%)	–	–	0.004
Work	–	27/40 (68%)	–	–	22/42 (52%)	–	–	0.16
Economy	–	27/40 (68%)	–	–	12/43 (28%)	–	–	<0.001
Leisure time activities	–	35/39 (90%)	–	–	25/43 (58%)	–	–	0.002
Forgo leisure-time activities because of symptoms	32 (78%)	29 (71%)	0.45	29 (54%)	18 (33%)	0.033	0.014	<0.001
Smoking habits:			0.90			0.10	0.68	0.11
Non-smokers	23 (56%)	25 (61%)		33 (65%)	42 (78%)			
Occasional smokers	5 (12%)	4 (10%)		6 (12%)	1 (2%)			
Daily-smokers	13 (32%)	12 (29%)		12 (24%)	11 (20%)			
Physical activity			0.76			0.89	0.89	0.96
Weekly	35 (85%)	34 (83%)		43 (84%)	45 (83%)			
Less than weekly	6 (15%)	7 (17%)		8 (16%)	9 (17%)			

Impact of the disease at baseline denotes expected impact, while at 24 months experienced impact.

*P values analyzed by Chi-square analysis, PSP = Patients with psychosocial problems, NPSP = Patients with no psychosocial problems.

Table 5 shows the results of age-adjusted logistic regression models on background factors at baseline, predicting experience of strong influence of RA at 24 months. Firstly, age- and sex-adjusted models for each factor are shown, and secondly a multivariate model, with adjustment for age and sex, and otherwise factors with p-value < 0.1 included. The only statistically significant factor was higher scores on HADS depression scale (p = 0.037). The model was tested for goodness-of-fit by Hosmer-Lemeshow’s test, p = 0.92.

**Table 5:** **Results of logistic regression, with strong impact of the disease at 24 months as outcome, in age-and sex-adjusted models for each tested variable, and multivariate model with adjustment for age, sex and educational level, but otherwise with factors with factors showing p-value < 0.1 included**

Factor	Sex and age-adjusted models	Multivariate model
	OR (95% CI)	OR (95% CI)
Age	0.99 (0.96–1.03)	1.01 (0.95–1.07)
Sex (women vs men)	1.01 (0.39–2.60)	0.61 (0.16–2.22)
Educational level:		
Compulsory school	1 (ref)	1 (ref)
High school	0.46 (0.14–1.51)	0.30 (0.07–1.31)
University	0.27 (0.08–0.85)	0.29 (0.07–1.21)
Employed	1 (ref)	
On early pension/long-term sick-leave	5.05 (1.49–17.15)	
Other	0.88 (0.26–2.99)	
Difficult financial situation	12.35 (1.47–103.72)	
Financial problems*	6.88 (1.39–34.08)	
PSP group	1 (ref)	1 (ref)
NPSP group	0.38 (0.23–0.63)	0.57 (0.31–1.05)
HAQ	2.53 (1.11–5.73)	
Patient-reported global perceived disease activity score	1.03 (1.01–1.04)	
Patient-reported pain	1.03 (1.01–1.05)	
HADS anxiety	1.28 (1.13–1.45)	
HADS depression	1.50 (1.24–1.80)	1.45 (1.18–1.77)
SOC score	0.93 (0.89–0.97)	
Coping strategies (GCQ):		
Self-trust	0.97 (0.95–1.00)	
Fatalism	1.03 (1.01–1.06)	
Problem focusing	1.00 (0.97–1.03)	
Resignation	1.02 (0.99–1.05)	
Cognitive revaluation	0.99 (0.97–1.01)	
Protest	1.03 (1.01–1.05)	
Social trust	1.00 (0.98–1.02)	
Isolation	1.06 (1.02–1.10)	
Minimization	0.96 (0.93–0.98)	
Intrusion	1.04 (1.01–1.07)	

Results presented as odds ratios (ORs), with 95% confidence intervals (95% CI).

*Have had difficulties paying bills during the last 12 months.

## Discussion

The main aim of this study was to investigate predictive factors for negative experienced effects of RA by the patients. We found that having higher scores on HADS depression was the only statistically significant predictor of perceived negative effects 24 months after diagnosis, being a consistent finding after adjustment and seemingly robust. Besides, differences between PSP patients and NPSP patients present at baseline were lasting over the two-year period.

The consisting differences between the PSP and NPSP groups over time, as well as the impact of the depression score on experienced impact of RA, are in line with findings by Evers et al., who found that stress-vulnerability factors seem to operate in the long run (Evers et al. 2003). Our results also was in line with findings from a recent study finding two subgroups of RA patients, with subjects characterized as belonging to one type (29%) being more prone to greater emotional expression and also to more time with depression (Tillmann et al. 2013). Another possibility could be that this psychosocial vulnerability could be a risk factor for developing RA, which not has been reported earlier, even if low socio-economic status has (Scott et al. 2011). No significant changes in social background variables over time between the two groups were found. However, the negative impact of the disease experienced after two years was stronger in both groups than expected at baseline. By contrast, both groups showed a significant improvement in clinical status over time, most likely an effect of successful medical RA treatment.

The psychological status regarding anxiety and depression was worse in PSP patients on both occasions, whereas the anxiety scores decreased significantly over time only in the NPSP group. We do not know to what extent the RA diagnosis affected the psychological status. The NPSP patients seemed to have a more successful way of dealing with the initial crisis reaction, supported by the decreased anxiety scores, but also the increased use of the coping strategy “cognitive revaluation”, and decreased use of “protest” and “intrusion”. However, the PSP patients might be psychologically more vulnerable, shown already at diagnosis as also found in other studies (Hyphantis et al. 2006). The PSP group also seemed to have less successful coping strategies, with more often use of “protest”, “isolation” and “intrusion”, and less of “minimization”.

Despite the improvement in clinical status, the scores for anxiety and depression did not decrease in the PSP group, which support the conclusion that what is important from the patient’s point of view is not the RA status as such but the psychological vulnerability, coping pattern and the social situation, which is at least partly in line with earlier findings (Curtis et al. 2005; Groarke et al. 2004). It appeared to be a discrepancy between the objectively measured disease activity on one hand, and the self-reported global sickness score, mood, and coping strategies on the other hand. As the presence of anxiety and depression may disturb the adaptation to RA, and possibly also the treatment at least as regards adherence, this underscores the need of finding markers of such problems early in the course of the disease (Isik et al. 2007).

Overall, PSP patients scored higher on negative coping strategies than did NPSP patients, as measured by GCQ. This is in line with findings of earlier studies that showed an association between these strategies and psychosocial and socio-economic problems (Gafvels et al. 2012; Rane et al. 2011; Gafvels and Wandell 2006,2007). Furthermore, studies on coping using other instruments have shown that passive coping, i.e. “efforts to avoid or deny the stressor” (Compas et al. 2012), which could also be regarded as a negative coping strategy, was associated with increased pain (Covic et al. 2000), while active coping, i.e. “efforts to act on the source of stress or one’s emotions” (Compas et al. 2012), could counteract the negative effect of perceived stress (Treharne et al. 2007). Furthermore, the adaptive strategies acceptance and problem solving are found to predict lower levels of psychopathology (Aldao and Nolen-Hoeksema 2012).

Low social support, especially support from workmates, which was reported more often by PSP than NPSP patients, has previously been shown to be associated with higher disease activity 3 years after diagnosis (Evers et al. 2003). This is in line with our finding with higher scores on self-reported sickness variables in PSP patients, in contrast to the lack of difference between the two groups in the disease activity score recorded by the rheumatologist.

Moreover, the patients who achieved their treatment goals of the psychosocial intervention experienced milder effects of the disease. RA patients with psychosocial problems due to the disease found it easier to achieve treatment goals than RA patients with problems due to other causes than the RA itself. This finding is important for tailoring psychosocial work to individual RA patient needs. Without the intervention the PSP patients could probably have been even worse off, but it is not possible to grade the magnitude of the effect due to lack of a control group not receiving this kind of intervention. An earlier meta-analysis found positive effects of psychosocial interventions in RA and osteoarthritis patients, with reductions in pain, anxiety, depression and psychological disability, and an improvement in active coping (Dixon et al. 2007).

### Strengths and limitations

The strength of the study is that it explored psychosocial problems in a sample of patients with RA, derived directly from everyday clinical practice, and the fact that we have used several well validated questionnaires to verify them. Besides, the differences between the two groups showed highly statistically significant results, and the final multivariate statistical model showed excellent goodness-of-fit.

One major limitation with this study is the small number of participants, which made it difficult to detect small differences between PSP and NPSP patients, especially over time. Thus, smaller but clinically relevant differences may not have adequately surfaced in the analyses. With regard to the size of the study groups, it was not possible, for ethical reasons, to have a sufficiently large group of RA patients with psychosocial problems not receiving any intervention as controls, and thus we cannot value the effect of the social worker’s intervention. Dividing the patients into two groups based on the presence or absence of psychosocial problems was done according to accepted clinical assessment criteria, i.e. whether the patients reported problems that required psychosocial intervention or not. However, the validity of this clinical classification was supported by the results of the self-reported questionnaires (HADS and SOC). The withdrawal rate was 19%, but there were no significant differences regarding age and sex between participants and non-participants, so the results could not be expected to be affected by this.

DAS28 could be problematic as a measurement of medical severity of RA, as part of the assessment is patient scoring of global disease impact on a VAS scale, and could be strongly affected by the patients’ psychosocial status. The use of the overall disease activity score assessed by rheumatologist could be questioned, but this score is a complement to DAS28 and other patient perceived disease activity, and is also reported to the SRQ Register. Besides, CRP values were not available. Furthermore, we did not have access to the specific treatment of the patients, but assume that this was in accordance with the treatment guidelines.

### Implications

Our findings, i.e. that having psychosocial problems already at diagnosis and higher scores on HADS depression scale, predicted a stronger negative impact of the disease at the 24-month follow-up, show the importance of recognizing emotional and social problems early in the course of RA (Hyphantis et al. 2006; Benka et al. 2013). Psychosocial problems increase the burden of the disease, including higher symptoms of anxiety and depression, and could affect the adherence to the medical treatment. Actually, without the psychosocial intervention in the PSP group the differences and impact of the disease would probably have been even more pronounced. This is an important knowledge for the rheumatologist, and a medical social worker should be a natural member of every RA team. The patients with psychosocial problems were identified by a semi-structured interview, and therefore it is important that medical social workers with knowledge about RA care should be available to support patients early in the course of the disease, rather than to use a questionnaire. Goals for further research could be to identify markers for a worse outcome, and to tailor specific actions. Psychosocial treatment goals seem to be easiest to achieve among RA patients with disease-related problems, and could preferably be treated by the medical social workers linked to the rheumatology department. Patients with other types of problems seem to need more extended support outside the hospital clinic. Besides, one could speculate in if the psychosocial vulnerability could be a risk factor for developing RA in genetically susceptible individuals, but this has to be analyzed in future studies.

## Electronic supplementary material

Additional file 1: **Interview form for medical social worker.** (DOC 28 KB)
