# Supplementary material for: What predicts negative effects of rheumatoid arthritis? A follow-up two years after diagnosis
Source: Springerplus. 2014 Feb 28;3:118. doi: 10.1186/2193-1801-3-118 (PMC3948188; doi:10.1186/2193-1801-3-118)
Supplement: Supplementary file 1 — Additional file 1: Interview form for medical social worker. (DOC 28 KB) [file 40064_2014_855_MOESM1_ESM.doc]

**Interview form for medical social worker**

Marital status: Unmarried

Married/co-habiting

Divorced

Widowed

Nationality: Swedish-born or immigrant (foreign-born)

Immigrantstatus: Schengen citizenship (EU)

Swedish citizenship/refugee

Swedish citizenship/other status

Residence/ refugee

Residence/ other status

Disabilities Yes (what); no

Profession according to Nordic classification of professions (NYK), and employment status

Education Compulsory school

High school

University

Other practical professional education

Partner’s profession and employment

Periods of sick-leave during the last 2 years (> 1 month)

On sick-leave now Yes (to which extent in %)/no

Utilities Driving license

Parking for the disabled

Transportation service for the disabled

Financial support for car

Own car

Access to a car

Disability benefits

Assistance at home

Personal assistance

Walking aids

Rehab compensation

Time-limited disability pension

Disability pension

Family/family members/sharing the household

Relationships within the family (partner, children, other)

Good; neither good nor bad; bad

Sexuality and partner relationship

Sexual relation

Sexual problems; if so, related to the disease?

Relationship to relatives/close friends

How many relatives could you get support from when needed?

How many friends could you get support from when needed?

Many; a few; none

Family of origin Members, and relationships between the members

Conditions during childhood, relationships to parents, siblings and important others

Current residence

Economy Good; acceptable; bad

Leisure time Practical interest

Cultural interest

Exercise/training

Sport performer

Seeing friends

Other interests

No special interests

Alcohol and tobacco

Tobacco: Yes (number of cigarettes daily); no

Alcohol: Yes (amount of alcohol in centiliters weekly); no

Other drugs: Yes (what?); no

Problems, abuse

Events of great importance for your life:

Positive (list; year)

Negative (list; year)

**Assessment by medical social worker**

Patient’s emotional status

Chaotic

Aggressive

Angry

Depressed

Anxious

Euphoric

Cheerful

Hopeful

Realistic

Other, what?

Patient’s experience of disease

Denying

Crisis, chaos

Stress, worried

Anger

Grief

Untouched

Acceptance

Other, what

Current problems or needs of community support according to the patient

Current problems or needs of community support according to the medical social worker

(Psychological treatment or support, or community support, i.e. assistance needs)

Further psycho-social investigation is needed

Treatment goals (for each problem):

Short term

Long term

Psycho-social treatment methods (for each problem):

Crisis management

Counselling

Motivational interviewing

Psycho-therapeutic intervention

Other, what?

Planned community support actions/contacts

Overall psycho-social assessment:

Patient needing psycho-social treatments and/or actions (PSP)

Patient not needing psycho-social treatments and/or actions (NPSP)
